# Electromagnetically induced transparency of a plasmonic metamaterial light absorber based on multilayered metallic nanoparticle sheets

**DOI:** 10.1038/srep36165

**Published:** 2016-11-08

**Authors:** Koichi Okamoto, Daisuke Tanaka, Ryo Degawa, Xinheng Li, Pangpang Wang, Sou Ryuzaki, Kaoru Tamada

**Affiliations:** 1 Institute for Materials Chemistry and Engineering, Kyushu University, Fukuoka, 819-0395, Japan; 2 Department of Electrical and Electronic Engineering, National Institute of Technology, Oita College, Oita, 870-0152, Japan; 3 Education Center for Global Leaders in Molecular Systems for Devices, Kyushu University, Fukuoka, 819-0395, Japan

## Abstract

In this study, we observed the peak splitting of absorption spectra for two-dimensional sheets of silver nanoparticles due to the electromagnetically induced transparency (EIT) effect. This unique optical phenomenon was observed for the multilayered nanosheets up to 20 layers on a metal substrate, while this phenomenon was not observed on a transparent substrate. The wavelength and intensities of the split peaks depend on the number of layers, and the experimental results were well reproduced by the calculation of the Transfer-Matrix method by employing the effective medium approximation. The Ag nanosheets used in this study can act as a plasmonic metamaterial light absorber, which has a such large oscillator strength. This phenomenon is a fundamental optical property of a thin film on a metal substrate but has never been observed because native materials do not have a large oscillator strength. This new type of EIT effect using a plasmonic metamaterial light absorber presents the potential for the development of future optic and photonic technologies.

Metal nanoparticles of silver, gold, or aluminium have large dipole oscillator strengths according to the localized surface plasmon (SP) modes and provide a highly enhanced electromagnetic field confined around the nanoparticles. The novel optical properties based on the localized SP resonance with light have been used for many applications. Recently, the “strong coupling” of the electromagnetic oscillator, especially using SP modes, has attracted a substantial amount of attention for providing quantum effect analogies. For example, the coupling between a SP mode and an exciton provides Rabi splitting^1^,^2^,^3^,^4^. Bellessa *et al*. used J-aggregate dye molecules and the propagation mode of SP with a total reflection setting with a prism and observed the splitting of the reflectance spectra, which reached an energy level of 180 meV^1^. A Rabi splitting energy of 250 meV was obtained by Dintinger *et al*. by employing the SP mode generated at subwavelength hole arrays^2^. Recently, it was reported that Rabi splitting reached 1 eV based on SP coupling using a metal-clad microcavity containing a thin organic semiconductor^4^.

The interferences of two electromagnetic modes cause a similar energy splitting, which is called normal mode splitting or electromagnetically induced transparency (EIT)^5^,^6^,^7^. Liu *et al*. reported a plasmonic analogy of the EIT effect using a combination of a gold bar as a dipole antenna and two gold wires as a quadrupole antenna^5^. The transparency window was observed in the broader absorption spectrum of the dipole antenna via coherent coupling with a sharper absorption spectrum of the quadrupole antenna. Using this transparency window, the absorption spectra were split into two spectra, and a nearly perfect transmission was achieved in the transparency window. The EIT effect was also observed using bilayered planar metamaterials that have a fish-scale pattern^6^. A similar interference phenomenon is called Fano resonance^8^,^9^,^10^ that occurs when the mode coupling is weaker than that in the EIT effect and when the resonance frequency of each mode is different. Mode coupling with reduced-symmetry cavity structures provides an asymmetric dip of the Fano resonance in the absorption spectrum. For example, Verellen *et al*. fabricated dolmen structures and ring disk structures by the E-beam lithography to observe the Fano resonances^9^. The resonance condition can be tuned by controlling the fabricated nanostructures. Self-assembled aggregations of metal nanoparticles were also used to obtain the Fano resonance by Fan *et al*. as an alternative to top-down processing^10^. The Fano dip in the absorption spectrum was observed even for the symmetric heptamer structure of Au nanoshells.

To achieve these interference effects in a strong coupling regime, both a narrow bandwidth of the resonance spectrum and a high value of the absorbance are required. In this study, we used multilayered metallic nanoparticle sheets up to 20 layers to achieve the EIT effect. Recently, we observed unusual absorption spectra that have a narrow bandwidth and high intensity by fabricating multilayered Ag nanoparticle sheet structures^11^,^12^. The optical property of the multilayered Ag nanoparticle sheets was attributed to the SP coupling of each nanoparticle over a wide area. The large electromagnetic fields are generated at the nanogap between the metal particles by the interaction of each localized SP mode^12^. This location is called the “Hot Spot” or “Hot Site”. The electromagnetic fields become moderately larger at the hot spot, and the absorption spectra become larger and broader. The hot spots were distributed over a wide surface of our nanosheets, and the collective interactions of each hot spot not only enhanced the interaction with light but also narrowed the resonance band. The resonance band can be tuned by the interparticle distance by changing the surfactant molecules of the nanoparticles^11^. We found that the resonance band of the multilayered Ag nanoparticle sheet on metal substrates could be easily tuned by adjusting the number of layers^12^. The intensity, wavelength, and linewidth of the absorption peak were changed by the number of layers and resulted in a colour change of the sample if they were placed on a metal substrate. We previously reported the unique optical properties of multilayered metallic nanoparticle sheets up to 5 layers^12^ and proposed several new applications, such as imaging^13^, colorimetric sensing^14^, and fluorescence controlling using Ag-Au nanosheets^15^. However, we had not noticed the peak splitting due to the EIT effect for multilayered metallic nanoparticle sheets until we have tried the layer deposition more than 5 layers.

## Results

### EIT effect of the multilayered AgMy nanosheets

**Figure 1:**
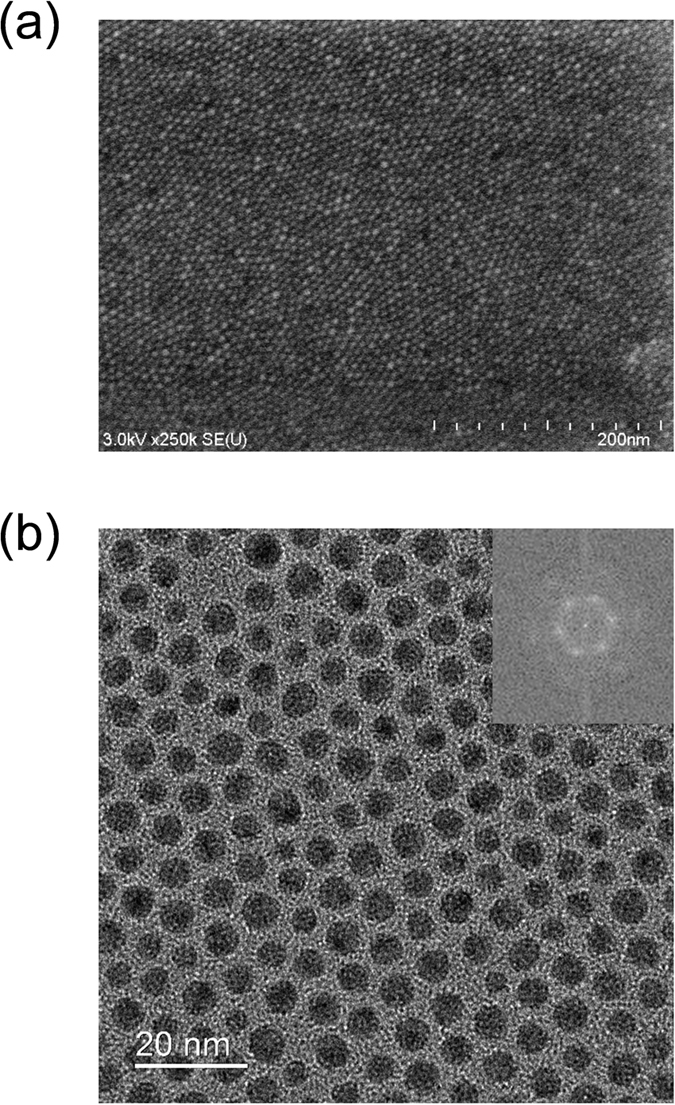
SEM and TEM images of the 2D nanosheet structure. SEM image (**a**) and TEM image (**b**) of the close-packed two-dimensional hexagonal structure of Ag nanoparticles. The Fourier transform image of this TEM image is also shown in the inset. The centre-to-centre distance was measured as 6.87 ± 0.77 nm.

Figure 1(a,b) shows the scanning electron microscope (SEM) image and the transmission electron microscope (TEM) image of the fabricated two-dimensional structure of the Ag nanoparticles capped with myristates (AgMy), respectively. The close-packed two-dimensional hexagonal structure was formed in this region. The Fourier transform image of the TEM image is also shown in the inset of Fig. 1(b). The centre-to-centre distance, namely the sum of the diameter and particle gap, was measured as 6.87 ± 0.77 nm. The atomic force microscopy (AFM) images of the AgMy sheet were also shown in Fig. S1. The multilayer was fabricated by the transfer of the 2D nanosheet to the substrate several times. Therefore, the particle position of each layer was not aligned.

**Figure 2:**
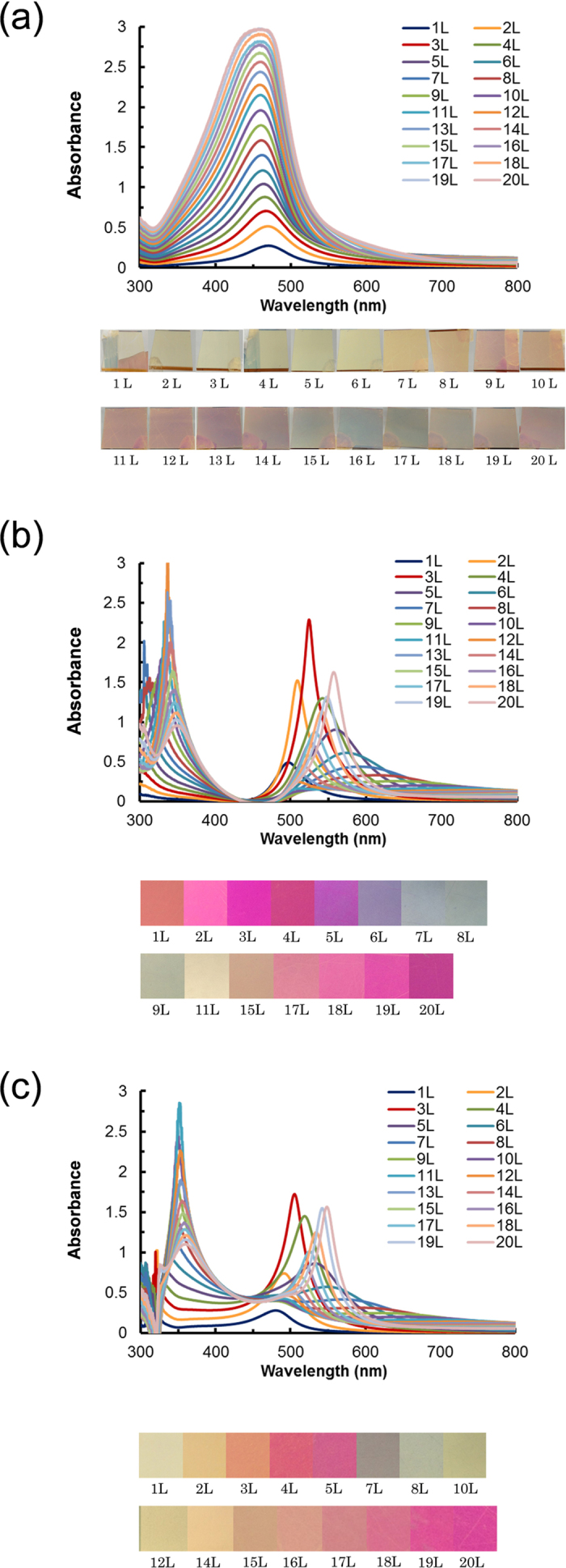
Experimental results of the absorption spectra. (**a**) Absorption spectra converted from the measured transmission spectra of the multilayered nanosheets on a quartz substrate. Transmission photographs of the samples taken on white paper are also shown. Absorption spectra converted from the measured reflection spectra of the multilayered nanosheets on gold (**b**) and silver (**c**) substrates. Reflection photographs of the samples are also shown.

Figure 2(a) shows the absorption spectra of the multilayered AgMy nanosheets on a quartz substrate converted from the measured transmission spectra. A single peak was observed at approximately 460 nm, and the peak intensities were increased by increasing the number of layers, thus increasing the thickness of the light-absorbing materials. The absorbance nearly reached 3 at 20 layers, suggesting that 99.9% of the incident light was absorbed in thin films that were only 180 nm thick. This high absorbance is difficult to obtain using natural materials. The peak wavelength appears slightly blue-shifted, and the spectra shape became broader as the number of layers increased. Transmission photographs of the samples taken on white paper are also shown. The yellowish colour of the surface originating with the Ag nanoparticles became deeper as the number of layers increased. The colour tones basically remained yellowish and turned into an intricate iridescent colour when the number of layers exceeded 10.

An interesting case occurred when using the metal substrates. Figure 2(b) shows the absorption spectra of the multilayered AgMy nanosheets on gold substrates. The peaks of the absorption spectra were clearly split into two peaks. This peak splitting became obvious when the number of layers exceeded 10. In our previous reports^12^, we could not find this peak splitting because we measured the absorption spectra of the multilayered AgMy nanosheet only up to 5 layers. We observed only one peak of the longer wavelength side and explained the complicated dependence of the absorption spectra on the number of layers as the combination of the red-shift and the nonlinear behavior^12^. We considered the origin of the red-shift as the local SP interaction enhanced by the inter-layer interactions. Here, we found that the more suitable explanation of the origin of the red-shift is due to the peak splitting by the EIT effect. The transparency dips were remarkable and nearly reached zero at approximately 460 nm, which was the original peak position observed on the quartz substrate. Perfect transparency was achieved at approximately 460 nm. Reflection photographs of the samples are also shown in Fig. 2(b). These colours dramatically changed with each layer number.

Similar results were obtained for the multilayered AgMy nanosheet on silver substrates in Fig. 2(c). A similar peak splitting phenomenon was observed on a silver substrate; however, the transparency dips did not reach zero at 460 nm. For the nanosheets on the silver substrate, perfect transparency was not achieved, and the minimum values of the absorption spectra were approximately 0.5. The different values of the minimum absorbance on each substrate were most likely due to the different reflection spectrum of the metal substrate.

In our previous study^12^, we reported that the inter-layer interactions of the SP modes of the Ag nanoparticles are not as effective because the interparticle distance in the vertical direction is larger than that of the lateral direction. Therefore, the observed phenomenon must be explained by not only the local interaction of each nanoparticle but also the macroscopic optical properties. The metal nanoparticles embedded in the media can be treated macroscopically as the composite dielectric materials. For example, the optical properties of the metal composite materials are given by the Maxwell-Garnet theory^16^. However, the unique optical properties of our multilayered nanosheets cannot be expressed by the Maxwell-Garnet theory because his theory is not applicable when the metallic nanoparticles are close to each other with a small separation gap at a high volume fraction and when the dipole interaction between adjacent nanoparticles is not negligible^11^,^12^. As mentioned above, the unique optical properties of AgMy nanosheets are attributed to the coupling of the SP modes of close-packed nanoparticles. The observed values of the absorbance (0.5–3.0) are very high, even when the thicknesses were only a few tens of nanometres. We proposed that the multilayered Ag nanoparticle sheets could be regarded as a dielectric material that has a very strong Lorentz oscillator strength by the effective medium approximation^12^. Thus, the multilayered AgMy nanosheet structures should be categorized as a plasmonic metamaterial^17^,^18^, exhibiting extraordinary macroscopic optical properties.

### Theoretical calculations using the Transfer-Matrix method

We attempted to reproduce the obtained EIT effects using the Transfer-Matrix (TM) method, which is a traditional method used to calculate propagations of plane waves. Recently, the TM method was used to analyse the Rabi splitting of the exciton-SP strong coupling^19^ and the EIT effects of the SP in waveguides^20^,^21^ and graphene^22^. For the TM calculations, we employed the effective medium approximation model for the AgMy nanosheets. The optical parameters of the Lorentz function model were estimated by the fitting of the absorption spectra of the multilayered AgMy nanosheets. The relative instantaneous permittivity (*ε*_∞_), plasma oscillation energy (*ℏω*_p_), resonance energy (*ℏω*_0_), and the damping energy (*ℏΓ*_p_) were obtained as 2.4, 2.63 eV, 2.75 eV, and 0.527 eV, respectively. The optical properties of this model are shown in Fig. S2. The extinction coefficients (*k*) of this model were ~1.3, and the refractive indexes (*n*) varied from 1 to 2.5 around the resonance frequency.

**Figure 3:**
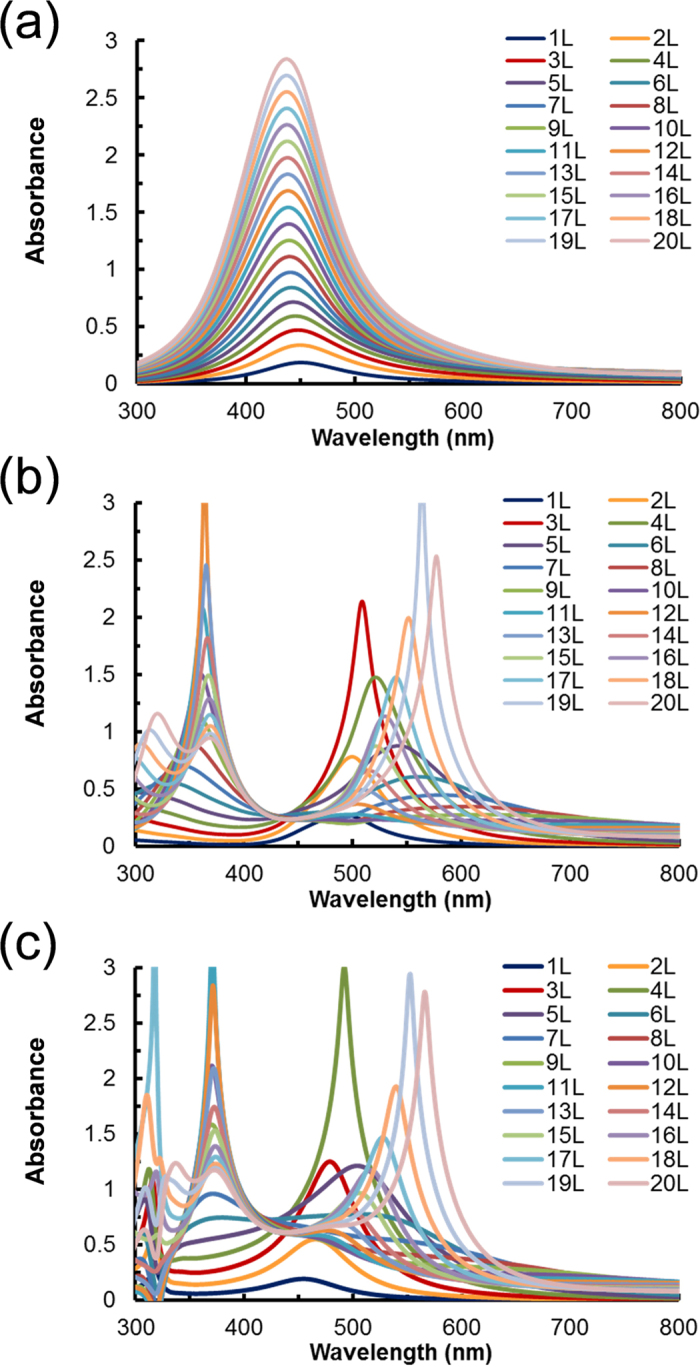
Calculation results of the absorption spectra. Calculation results of the Transfer-Matrix (TM) method for the absorption spectra converted from the transmission spectra on quartz (**a**) and the absorption spectra converted from the reflection spectra on gold (**b**) and silver (**c**) of thin films with the effective medium approximation model. The thickness varied from 9 nm (equivalent to 1 layer) to 180 nm (equivalent to 20 layers).

The calculated results for the thickness dependence of the absorption spectra converted from the transmission spectra on quartz and the absorption spectra converted from the reflection spectra on gold and silver are shown in Fig. 3(a–c), respectively. The thickness varied from 9 nm (equivalent to 1 layer) to 180 nm (equivalent to 20 layers). The splitting of the peaks on the metal substrates was well reproduced, while only one peak was obtained on the quartz substrates with a refractive index *n* = 1.5. These results suggest that the obtained unique optical properties can be explained based on the classical electrodynamic theory. The metal nanoparticles were used in this study to obtain strong extinction coefficients. However, the local plasmonic interaction of each particle did not need to be considered to reproduce the EIT effect except defining the *n* and *k* values as the initial parameters.

To evaluate a reliability of the calculated results by the TM method, we conducted a finite-difference time-domain (FDTD) simulation using the model with the Ag nanoparticle structures (Fig. S3). A similar peak splitting behaviour was also obtained by the FDTD simulations with the nanoparticles model; however, the calculated spectra were not in good agreement with the experimental spectra. The spectra calculated by the TM calculations with the effective medium approximation showed a much better reproduction of the experimental spectra. We used a regularly well-aligned metal nanoparticle structure with periodic boundary conditions for the model of the FDTD simulation [Fig. S3(a)]. In contrast, the actual structures of the multilayered AgMy nanosheets were not aligned in the vertical directions. The AgMy nanosheet has a hexagonal regular structure for a portion of the lateral directions; however, it has grain boundaries within a few hundred nanometres. Therefore, the alignment of the Ag nanoparticles in AgMy nanosheets was not very regular or periodic. For these non-regular structures, the effective medium approximation is better at reproducing their optical properties. We also confirmed that the absorption spectra calculated by the FDTD simulation were similar to the TM results when the same model with the effective medium approximation was used (Fig. S4). This result suggests that the TM method can sufficiently analyse the electromagnetic properties of the multilayered Ag nanoparticle sheet structures, even though the TM method is much simpler than the FDTD method.

We also calculated the dispersion diagram of light in the AgMy sheet using the FDTD method with the effective medium approximation model (Fig. S5). We found that the group velocities of the lights obtained by the slopes of these plots show negative values at 2.5~3.0 eV, which eventually agree with the transparency window regions.

### Behaviour and Mechanism of the EIT effect

**Figure 4:**
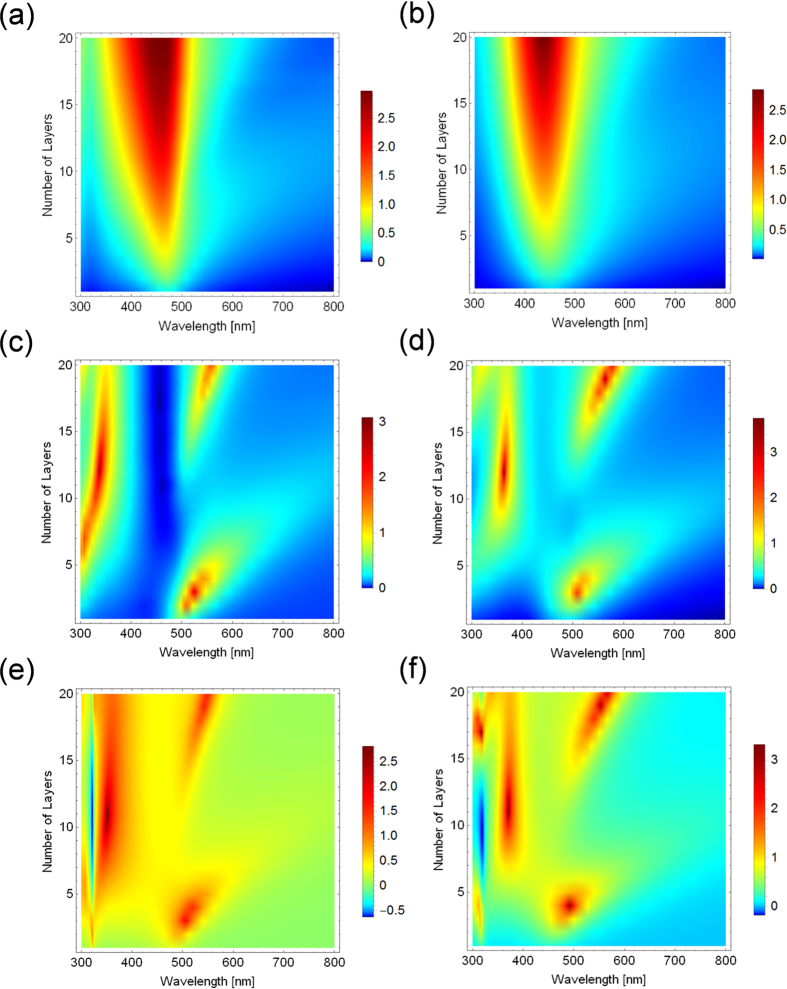
Dependence of the absorption spectra on the number of layers. Three dimensional representation of the dependence of the experimentally obtained absorption spectra on the number of layers on quartz (**a**) gold (**c**) and silver (**e**) substrates and the calculated spectra on quartz (**b**) gold (**d**) and silver (**f**) substrates.

To elucidate the origin and mechanism of the EIT effect observed in this study, we plotted a three dimensional representation of the dependence of the absorption spectra on the number of layers in Fig. 4. The dependence of the peak position, intensities, linewidth, and peak splitting behaviour of the absorption spectra against the number of layers can be clearly observed in these plots. The experimental results and the calculated results on each substrate were in good agreement each other. As mentioned above, the peak positions were blue-shifted as the number of layers on quartz increased. It is known that if the metal nanoparticles were arrayed in the lateral direction and the dipole modes interacted in the lateral direction, the resonant spectra should be red-shifted. However, if the metal nanoparticles were arrayed in the vertical direction and the dipole modes interacted in the lateral direction, the resonant spectra should be blue-shifted^23^. This phenomenon is similar to that for the J-aggregates in which the lateral interaction of the dipole causes a red-shift, while for the H-aggregates, the vertical interaction of the dipole causes a blue-shift^24^. The multilayered nanosheets used in this study exhibit a characteristic of arrayed nanoparticles in both the lateral and vertical directions especially when the layer number increased. This behaviour was also well reproduced by the calculations. In contrast, the absorption peaks were split into a higher-energy peak and a lower-energy peak on the metal substrates. The higher-energy peaks were observed with more than 7 layers, reaching their maximum values at approximately 10–12 layers, and this value decreased as the number of layers increased. On the other hand, the lower-energy peaks have two maximum values at approximately 3 layers and 20 layers, which disappeared at approximately 10–12 layers. When one of the two peaks has the maximum value, the other peak has the minimum value. These behaviours appear to be repeating as the number of layers increases. The periodic response of the peak intensity and wavelength according to the thickness of the material may be due to the interaction with the Fabry Perot resonance mode (Fig. S6). The detailed mechanism of this behaviour is still not completely clear, although the TM calculations based on the traditional electromagnetic theory could well reproduce the behaviour. The splitting energy reached 1.2 eV, which is as large as the highest reported values of the Rabi splitting^4^,^25^,^26^. This very large splitting energy is also one of the unique properties of our systems.

Because the EIT effect is based on the interference between two modes, it generally requires two types of nanostructures. The dipole oscillator and the quadrupole oscillator have often been fabricated by nano-lithography. A dipole oscillator with a symmetric structure, such as a metal sphere, wire, or rod, is the bright mode, producing a broad resonance peak. On the other hand, the quadrupole oscillator with asymmetric structures, such as a metal semi-sphere, crescent, wire pair, or double ring, is the dark mode, producing a sharp resonance peak because the interaction with light is weak. The interference of two modes can be generated when the resonance frequencies of the two modes are close. The EIT effect is observed when the peak frequencies of the two resonance spectra are the same, while the Fano resonance is observed when the two peak frequencies are different. In both cases, two types of nanostructures are required. However, in the case of this study, the sample does not have nanostructures; it is only a thin film on a metal substrate. We must explain how the interference effects can be feasible using this structure-less film.

The key to understand the mechanism is metal substrates required for this phenomenon. One of the possible mechanisms is the mode interaction through the mirror image generated in the metal substrate. When the excitation beam was irradiated to the oscillation media near the metal substrate, the charge oscillation in the metal substrate was induced by the mirror image effect. The phase of the oscillation in the mirror image is inverted to that of the oscillation of the excitation beam. If the phases of the two oscillations are the same, the interfacial event is the same as illuminating a film with double the thickness. On the other hand, if the phases of the two oscillations are opposite, the event should be the same as if the two different light-absorbing media exist. The dipole oscillator on the metal substrate can interact with the mirror image of the dipole oscillator. This interaction with the mirror image based on the Coulomb interaction should be long range and effective to the entire film thickness used in this study, which is up to 180 nm. for the sample thickness used in this study up to 180 nm. The interaction of these two oscillators, which has the opposite phase, generates a quadrupole oscillator with the same frequency. The interference of the dipole mode and the quadrupole mode causes the EIT effect and the splitting of the absorption spectra.

**Figure 5:**
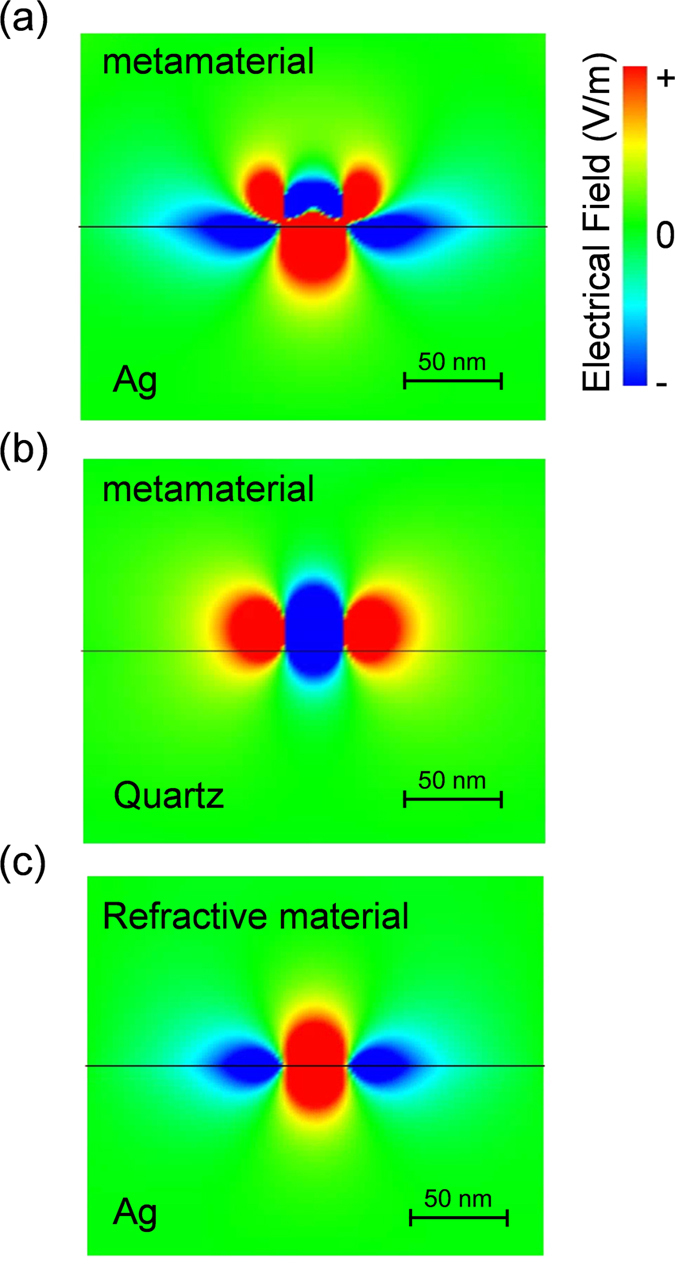
Electrical field distribution at the interface calculated using the FDTD method. (**a**) Electrical field distribution at the metal substrate calculated using the FDTD method with the effective medium approximation model for the metamaterials on a Ag substrate by exciting with a 30-nm point dipole located 10 nm from the metal substrate. (**b**) Reference calculation for the same sample on quartz. (**c**) Reference calculation for the model with the same refractive index and a zero absorbance.

To demonstrate the interference of the dipole mode and the quadrupole mode, we calculated the electrical field distribution at the interface of the metamaterial light absorber and the metal substrate. Figure 5 shows the electrical field distribution at the interface of dielectric film and the metal substrate, where the same effective medium approximation model as that used in Figs 3 and S4 was utilized for the FDTD calculation. We introduced 30-nm point light source at the 10 nm from the substrate and observed the electrical field distribution around the substrate. Figure 5(a) clearly shows the interaction with the mirror image on the Ag substrate and the formation of the quadrupole mode at the metal interface. The quadrupole modes were not observed on quartz [Fig. 5(b)] or film with a zero absorbance [Fig. 5(c)]. These results support our assumption that the interaction between the dipole and quadrupole induced the observed EIT effect. It was difficult to observe clear images of the electromagnetic modes when we used the plane wave excitations.

Recently, interactions of the electromagnetic modes through the mirror image were reported for silicon nanoparticles on a metal substrate^27^,^28^. Xifré-Pérez *et al*. demonstrated for the first time that the mirror image interaction of dielectric nanocavities placed near a flat metallic mirror causes a large amplification of the optical response. They reported that the optical interaction of the nanocavity with its mirror image is similar to that of two twin nanocavities in close contact, and the strong coupling through the mirror image leads to mode splitting^27^. Huang *et al*. reported that the Si nanoparticle dipole interaction with the mirror image results in two peaks for both the electric and magnetic resonances^28^. Mode coupling through the mirror image effect for structure-less thin films on metal substrates is the simplest structure that can induce the EIT effect, although it has not yet been reported.

## Discussion

**Figure 6:**
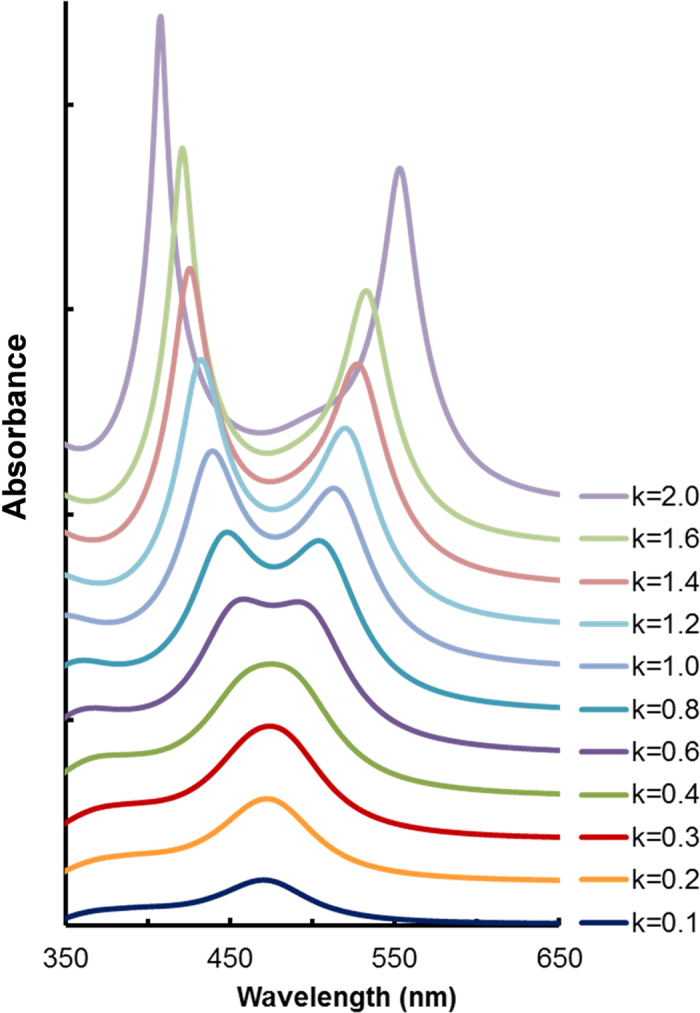
Conditions causing the EIT effect. TM calculations for the absorption spectra of the 135-nm-thick thin films of the Lorentz oscillator model on silver. Splitting of the absorption spectra depends on the extinction coefficient, namely, the oscillator strength.

To evaluate the required optical properties to induce this phenomenon based on the EIT effect, we calculated the absorption spectra of the same Lorentz oscillator model with various extinction coefficients (*k*). Figure 6 shows the obtained *k* dependence of the absorption spectra of this model with a 135-nm-thick film on silver. The absorption peak was a single peak under the condition of *k *= 0, and it began to split into two peaks as the *k* values increased.

Figure 6 suggests that the extinction coefficient must be larger than 0.6 to cause the peak splitting by the EIT effect. This result suggests that the observed EIT effect is feasible using the other materials if they have a sharp and strong oscillator strength with *k* > 0.6. Semiconductor and oxide materials have a large *k* value in the wavelength region shorter than the bandgap energy; however, the absorption spectra are very broad due to the band transition of electrons. On the other hand, organic molecules have absorption spectra with narrow bands, but the *k* values are not sufficiently large. The value of *k* is given by the molar extinction coefficient (*ε*/dm^3^mol^−1^cm^−1^) of the organic molecule as *k* = *εc*λ/4πlog_10_ *e*, where *c* and *λ* are the molar concentration and the wavelength of the irradiated light, respectively. The *k* values of typical organic molecules are lower than 0.001 because *ε* = 10^4^~10^5^ dm^3^mol^−1^cm^−1^. Therefore, this phenomenon had not been observed prior to this study using the typical materials.

Metamaterials that have artificial structures enable tuning of the optical properties and obtaining strong and narrow absorptions. In many cases, metamaterials have been fabricated using top-down nanotechnologies, such as E-beam lithography. However, these nanostructures are not suitable for application to wider areas of samples and to flexibly tune the coupling modes. The multilayered nanosheets used in this study can act as the plasmonic metamaterial, and we can tune the optical properties by changing the species, shape, size, and interparticle distance of the metal particles. We defined our multilayered nanosheets as plasmonic metamaterial light absorbers because it can provide a large absorbance.

Currently, the tunable EIT effect has been expected to have great potential for the development of new applications, such as single photon engineering^29^, slow light^30^, inversionless lasing^31^, and optical quantum information processing^32^. The plasmonic metamaterials presented in this study introduce a useful method for the flexible tuning of the EIT effects by adjusting the thickness on the metal substrate. We believe that the new techniques based on the plasmonic metamaterial light absorbers may develop into new platform technologies for optics and photonics.

## Methods

The Ag nanoparticles capped with myristates (AgMy) were synthesized by the thermal reduction of a silver acetate precursor in the melt of myristic acid, as previously described^33^. The diameter of the silver core was approximately 5 nm. Stable, close-packed two-dimensional sheet structures of AgMy were fabricated at an air–water interface via hydrophobic interactions in a Langmuir Blodgett (LB) trough (KSV NIMA small trough, Biolin Scientific, Sweden) at room temperature^11^,^12^,^13^,^14^,^15^,^34^. The AgMy nanosheet was transferred via the Langmuir–Schaefer (LS) technique onto hexamethyldisilazane (HMDS)-treated quartz substrates or dodecane thiol-functionalized thermally evaporated gold or silver thin films (200-nm thick) on quartz. The multilayered structure of the AgMy nanosheets was fabricated by transferring the nanosheets to the substrate several times.

The 2D nanostructure of the AgMy sheet was characterized using scanning electron microscope (SEM) (HITACHI SU8000). The AgMy film was transferred onto a SiO_2_ substrate which was treated with hexamethyldisilazane (HMDS), and observed with an accelerating voltage of 3.0 kV. The high-magnification image of the AgMy film was observed by using transmission electron microscopy (TEM) (JEOL JEM-ARM200F). The AgMy film was prepared on the pristine surface of a TEM grid (U1015: EM Japan) by LS technique, and observed with an accelerating voltage of 200 kV.

Transmittance and reflectance spectra were measured using a UV–vis spectrometer (UV-1800, Shimadzu, Japan) and were converted to absorption spectra, respectively. The contribution of the light scattering of the AgMy nanosheet must be very small because the diameters of the Ag nanoparticles (5 nm) were much smaller than the wavelength of the incident light. Therefore, the extinction spectra must include only the contribution of the absorption spectra. The literature values of the optical properties (refractive index and extinction coefficient) of gold and silver reported by Johnson and Christy^35^ were used for the TM calculations and FDTD simulations.

## Additional Information

**How to cite this article**: Okamoto, K. *et al*. Electromagnetically induced transparency of a plasmonic metamaterial light absorber based on multilayered metallic nanoparticle sheets. *Sci. Rep.* **6**, 36165; doi: 10.1038/srep36165 (2016).

**Publisher’s note:** Springer Nature remains neutral with regard to jurisdictional claims in published maps and institutional affiliations.

## Supplementary Material

Supplementary Information
