# Supplementary material for: Electromagnetically induced transparency of a plasmonic metamaterial light absorber based on multilayered metallic nanoparticle sheets
Source: Sci Rep. 2016 Nov 8;6:36165. doi: 10.1038/srep36165 (PMC5099917; doi:10.1038/srep36165)
Supplement: Supplementary Information [file srep36165-s1.pdf]

Supplementary Information

Electromagnetically induced transparency of a plasmonic metamaterial light absorber based on multilayered metallic nanoparticle sheets

Koichi Okamoto[1]*, Daisuke Tanaka[2], Ryo Degawa[1], Xinheng Li[1], Pangpang Wang[3], Sou Ryuzaki[1], and Kaoru Tamada[1]*

[1]Institute for Materials Chemistry and Engineering, Kyushu University, Fukuoka, 819-0395, Japan

[2]Department of Electrical and Electronic Engineering, National Institute of Technology, Oita College, Oita, 870-0152, Japan

[3]Education Center for Global Leaders in Molecular Systems for Devices, Kyushu University, Fukuoka, 819-0395, Japan

The fabricated two-dimensional structure of the Ag nanoparticles capped with myristates (AgMy) sheet was characterized using atomic force microscopy (AFM) (Asylum Cypher). The AgMy film was transferred onto a graphite substrate. A silicon probe AC160TS (Olympus) with a spring constant of ~26 Nm^{-1} and a resonant frequency of 300 kHz was used with tapping mode. **Fig. S1(a)** shows the AFM image of the surface morphology of the AgMy. The close-packed two-dimensional hexagonal structure was clearly observed in this region. **Fig. S1(b)** shows the AFM image with larger area and the line profile of the height. The thickness of the AgMy monolayer was measured as ~8 nm. That value is reasonable because the diameter of the Ag nanoparticle is ~5 nm and the length of the surfactant molecule is ~2 nm. We concluded that the single layer of the AgMy sheet has been successfully fabricated by the Langmuir–Schaefer (LS) technique.

For the theoretical calculations, we used the effective medium approximation model for the AgMy nanosheets. **Fig. S2(a)** shows the refractive indexes and the extinction coefficients of this model. The optical parameters of the Lorentz function model were obtained by the fitting of the absorption spectra of the multilayered AgMy nanosheets. **Fig. S2(b)** shows the absorption spectra of this model with 135-nm-thick thin films calculated by the Transfer-Matrix (TM) method. If the thin film was located on the quartz substrate with refractive index = 1.5, then the absorption spectrum showed the normal lorentzian shape with one peak. The absorption spectra were split into two peaks by the

EIT effect when the thin film was on the gold substrate.

To evaluate the calculated results of the TM method, we conducted finite difference time domain (FDTD) simulations using commercial software (Poynting for Optics, Fujitsu, Japan). The periodic boundary condition was set up in the X and Y directions, while a perfectly matched layer-absorbing boundary condition was set in the Z direction. A pulsed light composed of a differential Gaussian function with an intensity of 1 V/m was used as an excitation source. The pulse width was set to 0.5 fs, which corresponds to an average frequency of approximately 600 THz (500 nm wavelength). A non-uniform mesh was employed with a grid size of 0.1 nm to 10 nm. The dielectric function of the silver was approximated by the Drude-Lorentz formula whose parameters were fitted using the same literature values employed in the main text.

A model of the multilayered Ag nanosheets based on the arrayed Ag spheres is shown in **Fig. S3(a)**. The obtained absorption spectra converted from the transmission spectra on quartz and the absorption spectra converted from the reflection spectra on silver are shown in **Fig. S3(b)** and **(c)**, respectively. The absorption peaks were split into two peaks on the Ag substrates, while only one peak was obtained on the quartz substrates. However, the calculated results and the experimental results shown in Fig. 2(c) were not in good agreement compared to the calculated results of the TM method shown in Fig. 3.

We also attempted to reproduce the same results using the strong Lorentz oscillation model for the FDTD simulations. The strong Lorentz oscillation model based on the effective medium approximation was the same model as that used for the TM calculation in Fig. 3. The obtained transmission absorption spectra on quartz and the reflection absorption spectra on silver are shown in **Fig. S4(a)** and **S4(b)**, respectively. For comparison, the calculated results by the TM method using the same model on silver [Fig. 3(c)] are replotted in Fig. S3(c). The FDTD results shown in Fig. S3(b) are similar to the TM results using the same model shown in **Fig. S4(c)**.

According to these results, we concluded that the effective medium approximation model is much suited than the aligned metal nanoparticles model for the multilayered AgMy nanosheet. We also concluded that the TM calculation shown in the main text with the effective medium approximation model can sufficiently analyse the electromagnetic properties of the multilayered Ag nanoparticle sheet structures.

We evaluated the slow light effect in the metamaterial light absorber using the dispersion diagram. **Fig. S5** shows the dispersion diagram of the 270-nm-thick metamaterial light absorber used in this study calculated by the FDTD method with the effective medium approximation model. Fig. S5 shows the abnormal dispersion around the transparency window regions on both quartz and metal substrate. The group velocities

of the lights are obtainable by the slopes of these plots. The group velocities show negative values at 2.5 ~ 3.0 eV which correspond to the transparency windows on metal and the maximum absorption on quartz. A nearly identical dispersion was obtained on the both substrate. The slow light effects should be due to the defined effective medium approximation model and should not be the direct cause of the observed peak splitting. We concluded that the primary reason for the obtained peak splitting should be due to the EIT by the interference of the dipole mode and the quadrupole mode at the metal interface, as previously described.

We also considered the reason for the periodic response of the peak intensity against the wavelength and film thickness shown in Fig. 4. This may be due to the interaction with the Fabry Perot resonance mode generated in the materials. The destructive or constructive interaction may cause the periodic response of the peak intensities, wavelength, and peak widths, depending on the material thickness.

In **Fig. S6(a)**,**(b)**, we compared the periodic behavior of the absorption spectra with half of the wavelength of the light in the metamaterial light absorber. We found that the period is agree to the half of the wavelength of mode in this material. We also calculated the similar layer number dependence of the absorption spectra for the model that has double the value of the refractive index and the same absorption coefficient, as shown in **Fig. S6(c)**. **Fig. S6(d)** shows that the period was halved and remained similar to half of the wavelength when the refractive index doubled. This calculation results revealed that the periodic behavior should be due to the Fabry Perot resonance mode in the materials. **Fig. S6(e)** shows the layer number dependence of the calculated absorption spectra with the model with the same dispersion of the refractive index with k = 0. We found that the similar periodic structure without transparency window. Therefore, we concluded that the obtained EIT effect is not directly related to the Fabry Perot resonance. The EIT effect required the higher k values of the materials, while the periodic structures in the spectra required the variation of the refractive index.

Fig. S1 AFM images of the 2D nanosheet structure.

(a) AFM image of the close-packed two-dimensional hexagonal structure of Ag nanoparticles. (b) AFM image and line profile of the height at the line marked region. The thickness of the AgMy monolayer was measured as ~8 nm.

Fig. S2 Model and result for the TM method

(a) Optical properties of the Lorentz function model with ε_∞=2.4, $\hbar\omega_p$=2.63 eV, $\hbar\omega_0$=2.75 eV, and $\hbar\Gamma_p$=0.527 eV. (b) Transfer-Matrix (TM) calculation of the absorption spectra of the Lorentz oscillator model of (a) with 135-nm-thick thin films on quartz and gold.

Fig. S3 Model and result for the FDTD method

(a) A model for the FDTD calculation of the multilayered Ag nanosheets. (b) FDTD calculation results for (b) absorption spectra converted from the transmission spectra of the multilayered nanosheets on quartz and (c) absorption spectra converted from the measured reflection spectra of the multilayered nanosheets on silver.

Fig. S4 Comparison of the TM method and the FDTD methods

FDTD calculation results for the thin films with the Lorentz oscillator model for the absorption spectra on quartz (a) and on silver (b), (c) TM calculation of the absorption spectra with the same model as that in (b).

Fig. S5 Dispersion diagram of the metamaterial light absorber

Dispersion diagram of the 270-nm-thick metamaterial light absorber used in this study on a Ag substrate calculated using the FDTD method with the effective medium approximation model used in Fig. S4 for the metamaterial light absorber on silver (a) and on quartz (b).

Fig. S6 Periodic behaviour of the layer number dependence of the absorption spectra.

Comparison of the layer number dependence of the absorption spectra obtained experimentally (a) and using the TM calculation (b) shown in Fig. 4(c), (d) with half of the wavelength at 550 nm. (c) Optical properties of the model with the same adsorption coefficients and double the refractive index. (d) Comparison of the layer number dependence of the calculated absorption spectra with the model (c) with half of the wavelength at 550 nm. (e) Layer number dependence of the calculated absorption spectra with the model with the same dispersion of the refractive index with $k = 0$. White dashed lines are guide of eyes to show the transparency window.

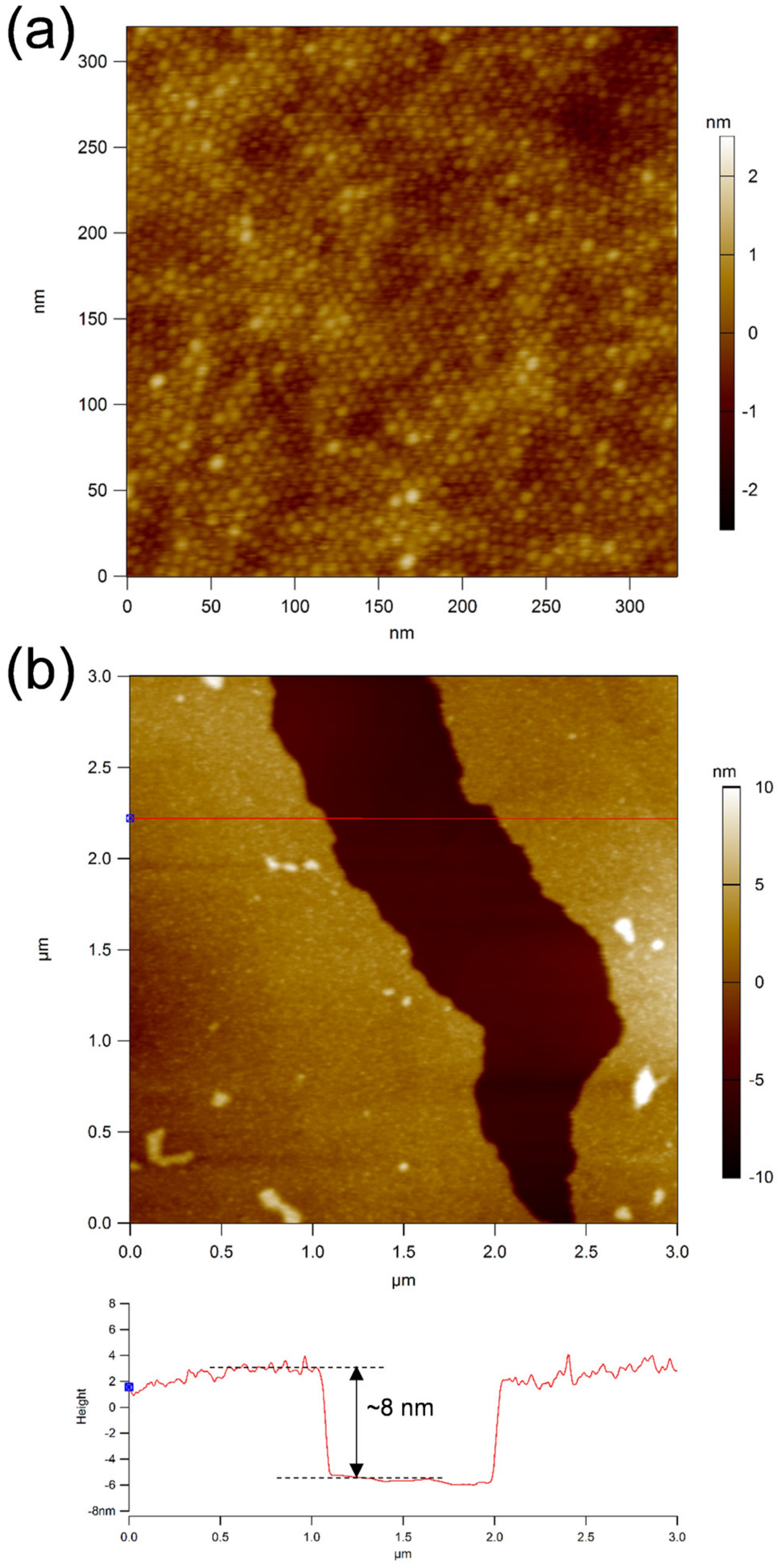

K. Okamoto et al. Fig. S1

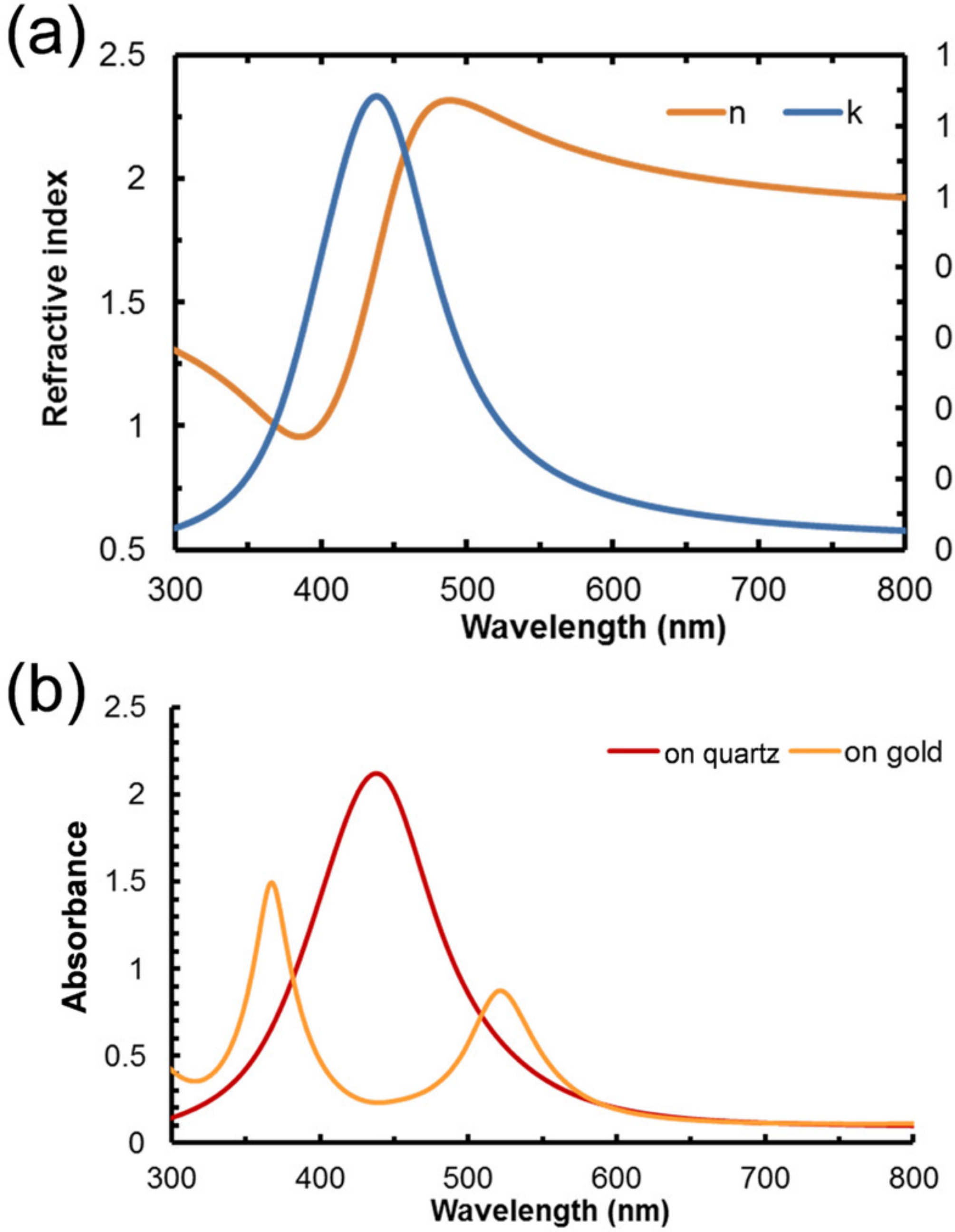

K. Okamoto et al. Fig. S2

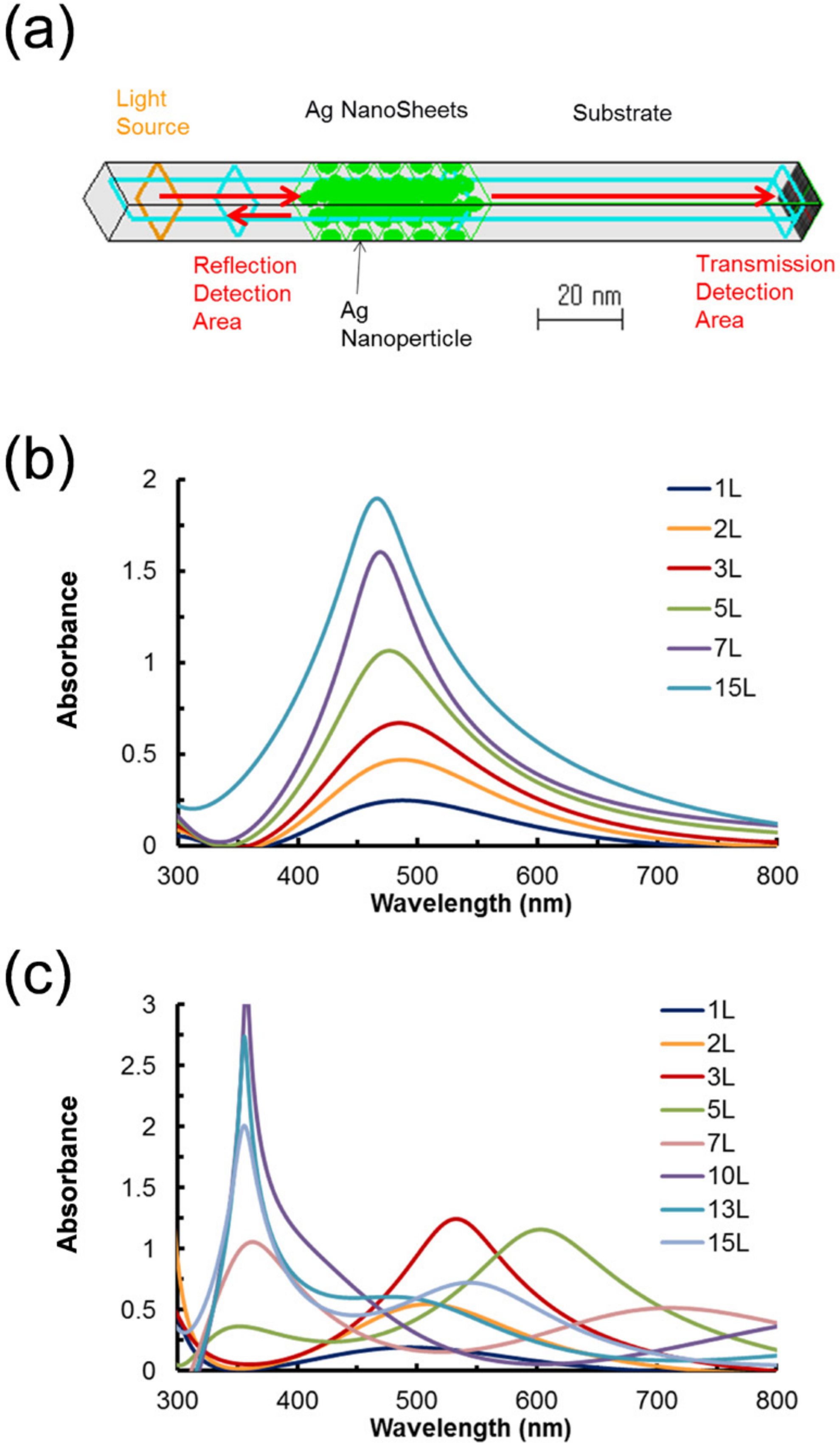

K. Okamoto et al. Fig. S3

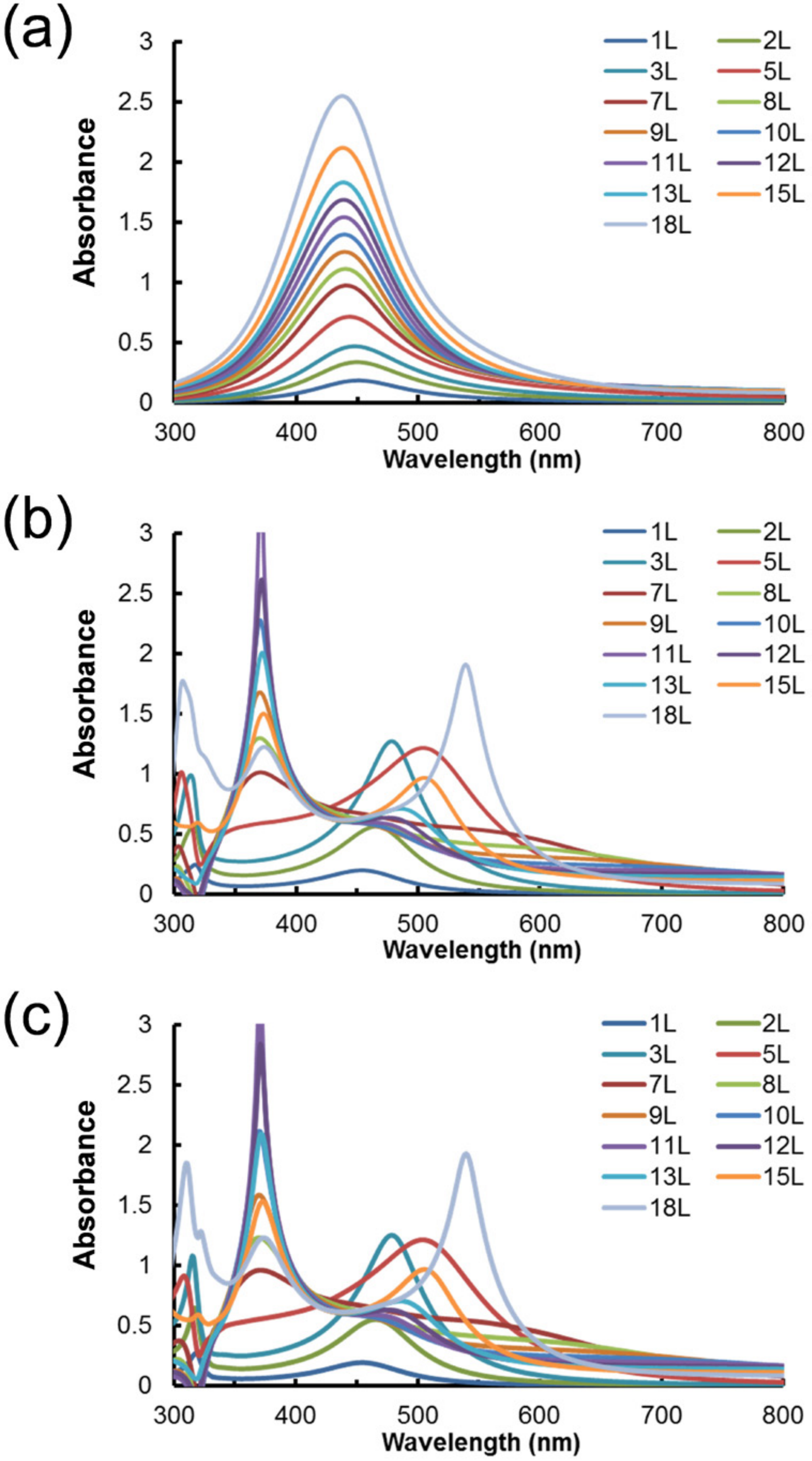

K. Okamoto et al. Fig. S4

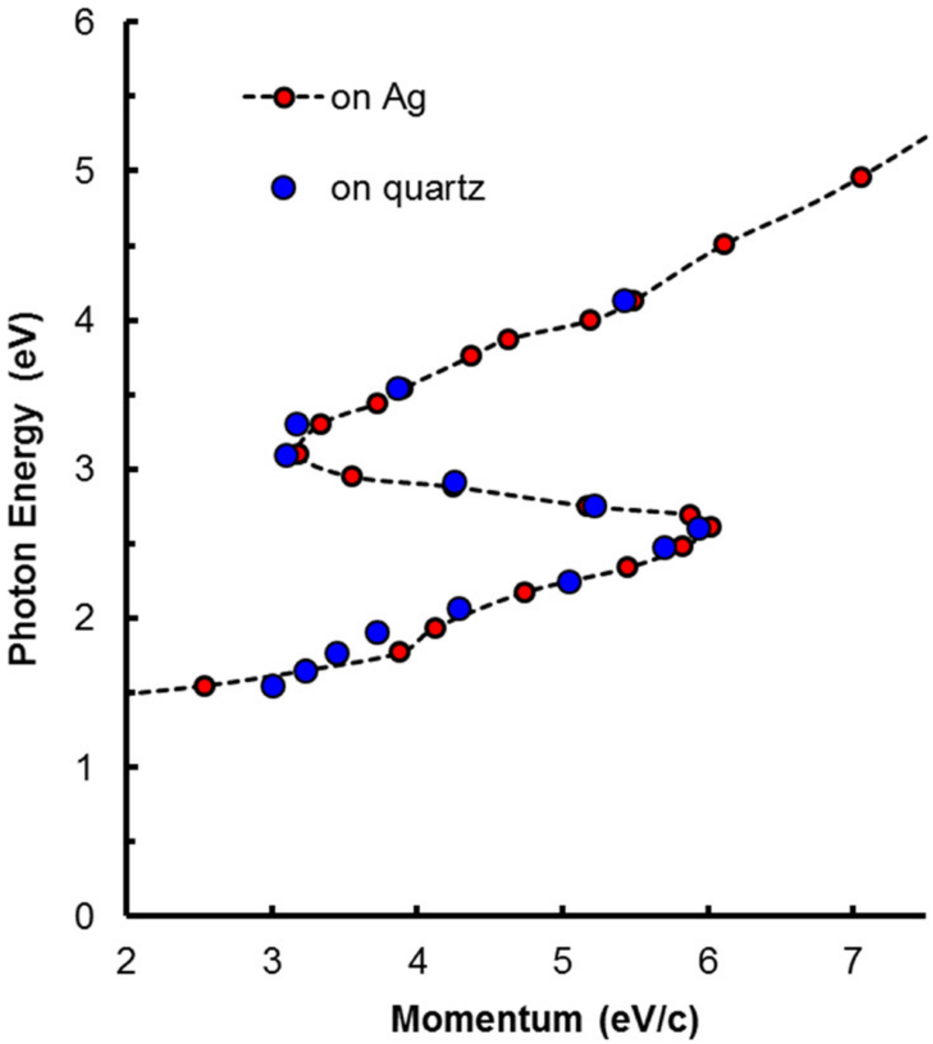

K. Okamoto et al. Fig. S5

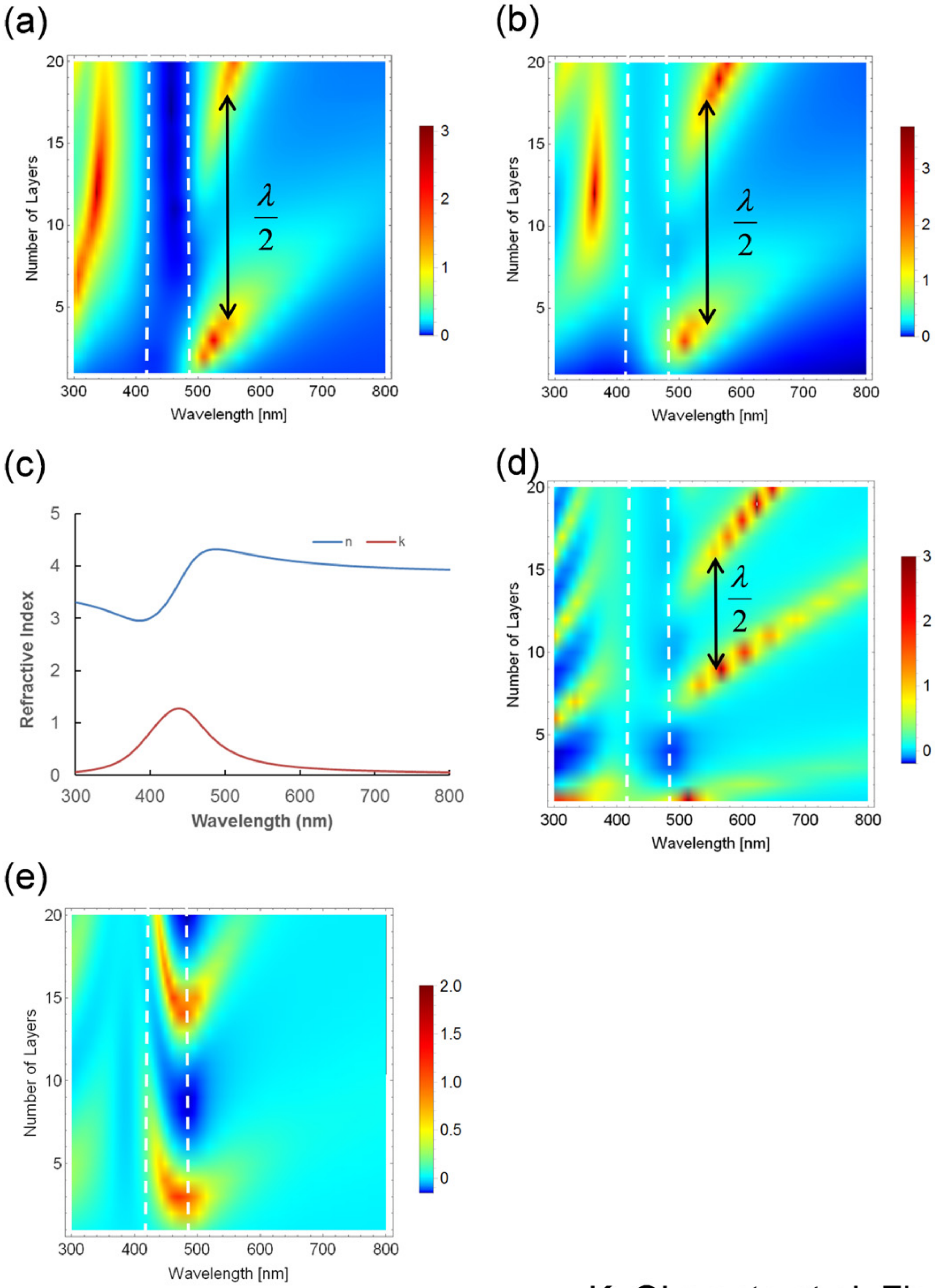

K. Okamoto et al. Fig. S6
